# Bartholows Cartwright Lectures

**Published:** 1881-02

**Authors:** 


BARTHOLOWS CARTWRIGHT LECTURES.—We regret that want of space denies us the pleasure of publishing these admirable Lectures, but we are consoled with the hope that they will be given to the profession in book form at an early period.

